# Regional differences in prostaglandin E_2_ metabolism in human colorectal cancer liver metastases

**DOI:** 10.1186/1471-2407-13-92

**Published:** 2013-02-26

**Authors:** Alastair L Young, Claire R Chalmers, Gillian Hawcroft, Sarah L Perry, Darren Treanor, Giles J Toogood, Pamela F Jones, Mark A Hull

**Affiliations:** 1 Section of Molecular Gastroenterology, Leeds Institute of Molecular Medicine, University of Leeds, St James’s University Hospital, Leeds LS9 7TF, UK; 2 Department of Hepatobiliary Surgery, St James’s University Hospital, Leeds LS9 7TF,UK; 3 Department of Pathology and Tumour Biology, Leeds Institute of Molecular Medicine, University of Leeds, Leeds LS9 7TF, UK

**Keywords:** Colorectal cancer, Epithelial-mesenchymal transition, Hypoxia, Liver metastasis, Prostaglandin

## Abstract

**Background:**

Prostaglandin (PG) E_2_ plays a critical role in colorectal cancer (CRC) progression, including epithelial-mesenchymal transition (EMT). Activity of the rate-limiting enzyme for PGE_2_ catabolism (15-hydroxyprostaglandin dehydrogenase [15-PGDH]) is dependent on availability of NAD+. We tested the hypothesis that there is intra-tumoral variability in PGE_2_ content, as well as in levels and activity of 15-PGDH, in human CRC liver metastases (CRCLM). To understand possible underlying mechanisms, we investigated the relationship between hypoxia, 15-PGDH and PGE_2_ in human CRC cells *in vitro*.

**Methods:**

Tissue from the periphery and centre of 20 human CRCLM was analysed for PGE_2_ levels, 15-PGDH and cyclooxygenase (COX)-2 expression, 15-PGDH activity, and NAD+/NADH levels. EMT of LIM1863 human CRC cells was induced by transforming growth factor (TGF) β.

**Results:**

PGE_2_ levels were significantly higher in the centre of CRCLM compared with peripheral tissue (P = 0.04). There were increased levels of 15-PGDH protein in the centre of CRCLM associated with reduced 15-PGDH activity and low NAD+/NADH levels. There was no significant heterogeneity in COX-2 protein expression. NAD+ availability controlled 15-PGDH activity in human CRC cells *in vitro*. Hypoxia induced 15-PGDH expression in human CRC cells and promoted EMT, in a similar manner to PGE_2_. Combined 15-PGDH expression and loss of membranous E-cadherin (EMT biomarker) were present in the centre of human CRCLM *in vivo*.

**Conclusions:**

There is significant intra-tumoral heterogeneity in PGE_2_ content, 15-PGDH activity and NAD+ availability in human CRCLM. Tumour micro-environment (including hypoxia)-driven differences in PGE_2_ metabolism should be targeted for novel treatment of advanced CRC.

## Background

Colorectal cancer (CRC) remains the third most common cancer worldwide with more than one million new cases reported in 2008 [1]. The liver is the most common site of CRC metastasis with 50-60% of CRC patients eventually developing liver disease [2]. Metastasis, in common with growth and invasion of established tumours, is dependent on tumour cells acquiring a migratory and invasive phenotype as part of a highly conserved cellular trans-differentiation programme, the epithelial-mesenchymal transition (EMT) [3,4]. Prostaglandins (PG), in particular PGE_2_, have previously been implicated in EMT of CRC cells [5].

Prostaglandins (PG) are fatty acid signaling molecules known to have a range of physiological functions including vascular homeostasis, reproduction and immune regulation [6]. PGE_2_ is the most abundant PG in the human colon and levels of PGE_2_ are increased in colorectal neoplasia compared with normal colorectum [7]. Elevated PGE_2_ levels are known to promote colorectal carcinogenesis at various stages, including CRC growth and metastasis [6]. Recently, PGE_2_ has been implicated in promotion of EMT *in vitro*[8]. PG G/H synthase (also known as cyclooxygenase [COX]) controls the rate-limiting step in PGE_2_ synthesis, upstream of PGE synthases [6]. There are two COX isoforms; the constitutive isoform COX-1 and the inducible isoform COX-2, which is up-regulated in CRC and is a putative target for anti-CRC therapy [9,10]. Nicotinamide adenine dinucleotide (NAD+)-linked 15-hydroxyprostaglandin dehydrogenase (15-PGDH) controls the rate-limiting step in PGE_2_ catabolism by conversion of PGE_2_ to 15-keto-PGE_2_ coupled to the reduction of NAD+ to NADH [11]. Initial studies suggested that 15-PGDH expression is reduced in bladder cancer, lung cancer and CRC compared with paired normal tissue and has tumour suppressor properties [12]. However, subsequent reports have highlighted elevated 15-PGDH expression in breast and ovarian cancer [12]. Moreover, there are conflicting data on 15-PGDH expression in gastric cancer [12]. Heterogeneity of 15-PGDH expression in human cancers may reflect tissue-specific differences in regulatory pathways upstream of 15-PGDH [12], but may also be related to sampling variation secondary to intra-tumoral genetic and micro-environmental influences on 15-PGDH expression [13,14].

There has been relatively little investigation of changes in 15-PGDH *activity*, as opposed to gene expression, in human cancer. Although NAD+, and its phosphorylated form NADP, act as critical hydride-accepting co-enzymes in cellular metabolism, NAD+ is also a substrate for three classes of NAD+ −consuming enzymes, which are the poly(ADP-ribose) polymerases (PARPs), cADP-ribose synthases and sirtuins, expression and activity of which can be altered in cancer cells [15,16]. Therefore, a valid hypothesis is that NAD+ availability is rate-limiting for 15-PGDH activity and PGE_2_ catabolism in CRC cells.

Regional hypoxia is common in many cancers including CRC [17], in which established markers of tumour hypoxia have been linked to worse prognosis [18]. Central tumour areas are believed to be more hypoxic than peripheral tumour tissue [19] as demonstrated in CRC liver metastases (CRCLM) by dynamic con-trast-enhanced magnetic resonance imaging [20] and immunohistochemistry (IHC) for carbonic anhydrase IX [21]. Hypoxia is associated with increased PGE_2_ production and release by several human cell types, including human CRC cells, *in vitro*[22-24]. This is believed to occur via induction of the COX-2-PGE synthase axis, with no change in 15-PGDH expression, although 15-PGDH activity and NAD+/NADH levels were not measured in these studies [23,25]. Expression of NAD+ −consuming enzymes such as SIRT1 is increased in hypoxic cells [26] and overall NAD+ levels have been demonstrated to be reduced in ischaemic tissue, as well as a reduction in the NAD+/NADH ratio [27].

Given the potential micro-environmental influence of hypoxia and co-factor availability on PGE_2_ metabolism, we tested the hypothesis that there are regional differences in PGE_2_ levels within human CRCLM, which are related to differential expression and activity of 15-PGDH and COX-2 within tumours. To this end, we collected and analysed human CRCLM tissue from peripheral and central areas of tumours in a systematic, protocol-driven manner, comparing our tissue findings with observations in human CRC cells *in vitro*, including those from the LIM1863 human CRC cell model of EMT.

## Methods

Detailed methodological descriptions are available in Additional file 1: *Methods*.

### Tissue collection

**Figure 1:**
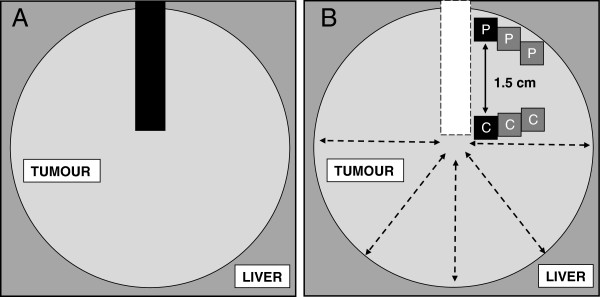
**Retrieval and processing of human CRCLM tissue.** Diagram showing the dissection method for obtaining CRCLM tissue (light grey) from a liver resection specimen (dark grey). **A**) A slice of tissue (dark rectangle) incorporating both central and peripheral tumour, as well as a rim of surrounding liver, was obtained for fixation in formalin and subsequent embedding in paraffin wax. **B**) Tumour orientation and the location of the centre of the tumour were confirmed with at least four other radial incisions (dashed arrows). Six peripheral tumour samples (each approximately 0.5 cm^3^) were taken from within 0.75 cm of the tumour margin (marked P) taking care not to include any liver parenchyma or tumour capsule. Similar sized central tumour samples (marked C; n = 6) were then taken from an area at least 1.5 cm from the peripheral samples. Samples were immediately transferred to ice for assay or into liquid nitrogen for storage at −80°C.

Approval for the study was obtained from the Leeds (East) Research Ethics Committee. Tissue was retrieved from 20 patients undergoing a first liver resection for CRCLM at the Hepatobiliary Unit at St James’s University Hospital, Leeds between March 2007 and April 2008. A minimum tumour diameter of 3.5 cm in all dimensions was required so that tissue from clearly defined central and peripheral regions could be obtained. Patients on regular (defined as more than two doses per week in the previous month) aspirin or non-aspirin non-steroidal anti-inflammatory drug (NSAID) therapy were excluded, as were any patients who had received any form of cytotoxic chemotherapy in the preceding three months. Fresh tumour tissue was dissected in the operating theatre according to a strict protocol (Figure 1) and samples were immediately placed on ice, prior to immediate further processing or analysis.

### PGE_2_ assay

Tissue PGE_2_ levels were measured using a competitive immunoassay (Amersham Biosciences). Total protein was measured using a Bradford protein assay kit (Bio-Rad Laboratories Ltd., Hemel Hempstead, UK). Data are presented as pg PGE_2_ per mg total protein. PGE_2_ levels in cell-conditioned medium are presented as pg per cell number.

### Immunohistochemistry

Immunohistochemistry (IHC) for 15-PGDH, COX-2 and E-cadherin was performed on 5 μm sections of formalin-fixed paraffin-embedded CRCLM tissue, which included peripheral and central tumour regions (Figure 1). COX-2 IHC was performed as previously described by the Hull laboratory [28] using a rabbit polyclonal antibody to COX-2 (IBL, Gunma, Japan). Immunohistochemistry for 15-PGDH and E-cadherin is described in Additional file 1*: Methods*. All slides were counterstained with haematoxylin. Negative controls were prepared by omission of the primary antibody.

### Quantitative immunohistochemistry analysis

A computerized scoring method was developed to ensure objectivity in selecting central and peripheral tumour regions of interest and to quantify immunoreactivity in each region of interest (see Additional file 1: *Methods* and Additional file 2: Figure S1). Immunostained slides were digitized using a Scanscope XT (Aperio, Vista, CA, USA) and then analysed using Imagescope (Aperio v8.2) software. The mean immunoreactivity for tumour cells in central and peripheral tumour regions was calculated for each tumour.

### Tissue microarray of primary CRC and CRCLM tissue

A tissue microarray (TMA) consisting of two replicates of each of three cores from both the centre and peripheral region of a primary CRC and a synchronous/metachronous CRCLM from 38 patients was constructed as described [29]. Immunohistochemistry for 15-PGDH was performed as described above and each core was scored for 15-PGDH immunoreactivity by two independent observers based on the intensity of cytoplasmic staining of tumour cells on a scale of 1–4 (see Additional file 3: Figure S2). There was excellent agreement between the observers (κ score 0.94). The median 15-PGDH score for each tumour region was derived from a maximum of 12 possible scores (3 cores each represented twice, independently scored by two observers) for each tumour area.

### Human cancer cell culture

HCA-7 human CRC cells were cultured as described [28]. LIM1863 human CRC cells were obtained from the Ludwig Institute (Parkville, Melbourne, Australia) and were cultured in the presence of 5% CO_2_ in RPMI 1640 with 5% (v/v) foetal calf serum (FCS; Invitrogen). EMT was induced in LIM1863 cells (Additional file 4: Figure S3) by 2 ng/ml transforming growth factor β (TGFβ; R&D Systems Europe Ltd., Abingdon, UK) [30]. MCF-7 human breast cancer cells were obtained from the European Collection of Cell Cultures and were cultured in RPMI 1640 (Sigma) with 5% (v/v) FCS. Cells were cultured in normoxic (20% O_2_) or hypoxic conditions (1% O_2_) in a Sanyo MCO-175 M incubator (Panasonic, Loughborough, UK) in pre-equillibrated media.

### 15-PGDH mRNA analysis by quantitative RT-PCR

Total RNA was extracted and reverse transcribed as previously described [31]. SYBR-Green™ real-time PCR was performed using an ABI 7700 sequence detection system using primers for 15-PGDH (forward, 5’-d(TAGTTGGATTCACACGCTCAGC); reverse, 5’-d(AAAGCCTGGACAAATGGCAT) and the ‘reference’ ribosomal protein gene 36B4, which is unresponsive to hypoxia (forward, 5’-d(GAAACTCTGCATTCTCGCTTCC); reverse, 5’-d(GATGCAACAGTTGGGTGCCA) [32]. Levels of 15-PGDH transcripts were quantified using the 2^-ΔCt^ method [33].

### 15-PGDH enzyme activity assay

15-PGDH enzyme activity in CRCLM tissue was measured as described [34]. In brief, tumour cell lysate was incubated with glutamate dehydrogenase (Sigma) in the presence of 1 nM [^3^H]-PGE_2_ and 1 μmol NAD+ (Sigma). Data are expressed as cpm per 100 mg/protein. Any values below the negative control (MCF-7 cell lysate heat-inactivated at 100°C for 15 minutes) were excluded. The detailed protocol is provided in Additional file 1*: Methods*.

### NAD^+^/NADH assay

Cell and tissue lysates were produced by mechanical disruption with a Dounce grinder followed by 2 freeze/thaw cycles (20 minutes at −80°C then 10 minutes at 25°C). Lysates were immediately passed through a 10 kDa molecular weight cut-off filter (Biovision, Milpitas, CA, USA). NAD+ (derived from total NAD minus NADH) and NADH concentrations were measured in peripheral and central CRCLM tissue, as well as in LIM 1863 human CRC cells and in MCF-7 human breast cancer cells, using an NAD+/NADH assay as per manufacturers’ instructions (Biovision).

### Immunofluorescence

Immunofluorescence was performed on methanol-fixed LIM1863 cells, using the same antibodies against 15-PGDH and E-cadherin used for tissue immunohistochemistry. Secondary antibodies used were donkey anti-rabbit, Alexa Fluor® 488 (for 15-PGDH) and goat anti-mouse Alexa Fluor® 594 (for E-cadherin; both Invitrogen). Cells were visualised using a Zeiss Axiostar microscope (Zeiss). Further detail is provided in Additional file 1: *Methods*.

### LIM 1863 human CRC EMT assay

LIM 1863 cells were cultured in 6-well plates pre-marked with a 12-square grid for orientation. Recombinant human TGFβ was added for 48 hours before imaging. The first 25 adherent colonies, identified by systematic scanning of the grid, were photographed on day 2 (Additional file 4: Figure S3) and their position in the grid recorded for repeat imaging after a further 4 days, if still adherent. NIS elements BR2.2 software (Nikon) was used to measure the area change in every adherent colony per well. The mean percentage area change between day 2 and day 6 was calculated and the mean value was derived from three separate wells per condition.

## Results

### PGE_2_ levels are higher in the central region of CRCLM relative to peripheral tumour tissue

**Figure 2:**
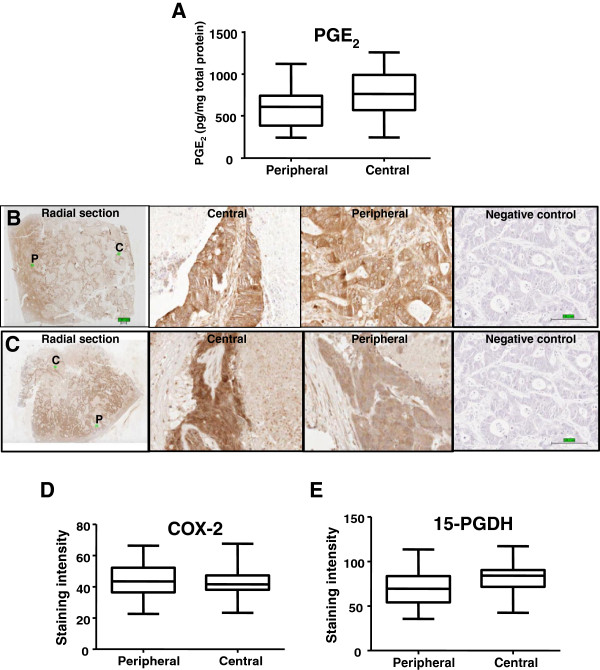
**Regional differences in PGE**_**2 **_**levels and 15-PGDH immunoreactivity in human CRCLM. A**) Box and whisker plot of the median, inter-quartile range and range of the PGE_2_ level in peripheral and central areas of CRCLM. Immunohistochemistry for COX-2 (**B**) and 15-PGDH (**C**) in human CRCLM. In each case, a low-power view of the whole section covering the radius of a CRCLM is shown (bar = 1 mm). Areas in the centre (**C**) and periphery (P) of the tumour are marked with a green square and are shown as the corresponding high power view, as well as a negative control section, for which the primary antibody was omitted (bar = 100 μm). **D**)-**E**) Box and whisker plots of the median, inter-quartile range and range of COX-2 immunoreactivity (**D**) and 15-PGDH immunoreactivity (**E**) in the peripheral and central area from 20 human CRCLM. Immunohistochemical staining in **D**) and **E**) is measured in arbitrary units on a scale of 0–220 derived from the Aperio Imagescope software.

Initial studies explored whether there was a difference in PGE_2_ content between different areas of CRCLM. The median PGE_2_ level in central and peripheral regions of CRCLM was 762 pg/mg protein and 603 pg/mg protein respectively (Figure 2A). PGE_2_ levels were increased in the central region of the tumour compared with the peripheral region in 14 of 20 CRCLMs (Additional file 5: Figure S4). There was a mean 26% (95% CI 1%-52%) higher PGE_2_ level in central tumour regions relative to paired peripheral tumour tissue (p = 0.04, Wilcoxon rank sum test).

### 15-PGDH protein levels are higher in central tumour regions relative to peripheral CRCLM tissue

Next, we investigated regional expression of the rate-limiting enzymes for PGE_2_ synthesis and catabolism. Representative IHC for COX-2 and 15-PGDH on CRCLM tissue is shown in Figure 2B-C. A median of 764 810 pixels per region were measured (range: 14,782 – 26,369,364). There was no significant difference between COX-2 staining intensity in cancer cells between paired peripheral and central tumour regions in CRCLMs (p = 0.80, Figure 2D and Additional file 5: Figure S4). However, there was significantly higher 15-PGDH immunoreactivity in cancer cells in the tumour centre relative to the cancer cells at the tumour periphery in 13 of 18 CRCLM (Figure 2E and Additional file 5: Figure S4). There was a mean 14% (95% CI 3%-27%) increase in 15-PGDH immunoreactivity in central tumour regions compared with paired peripheral tissue (p = 0.04). Differential regional expression of 15-PGDH in CRCLM was also observed using an independent tissue microarray consisting of tissue cores from the centre and periphery of 38 CRCLM (Additional file 3: Figure S2). Importantly, no difference in 15-PGDH immunoreactivity between central and peripheral areas was observed in the tissue microarray of primary CRCs from the same patients as the CRCLM (Additional file 3: Figure S2) suggesting that this phenomenon is specific to CRCLM, as opposed to primary tumours.

**Figure 3:**
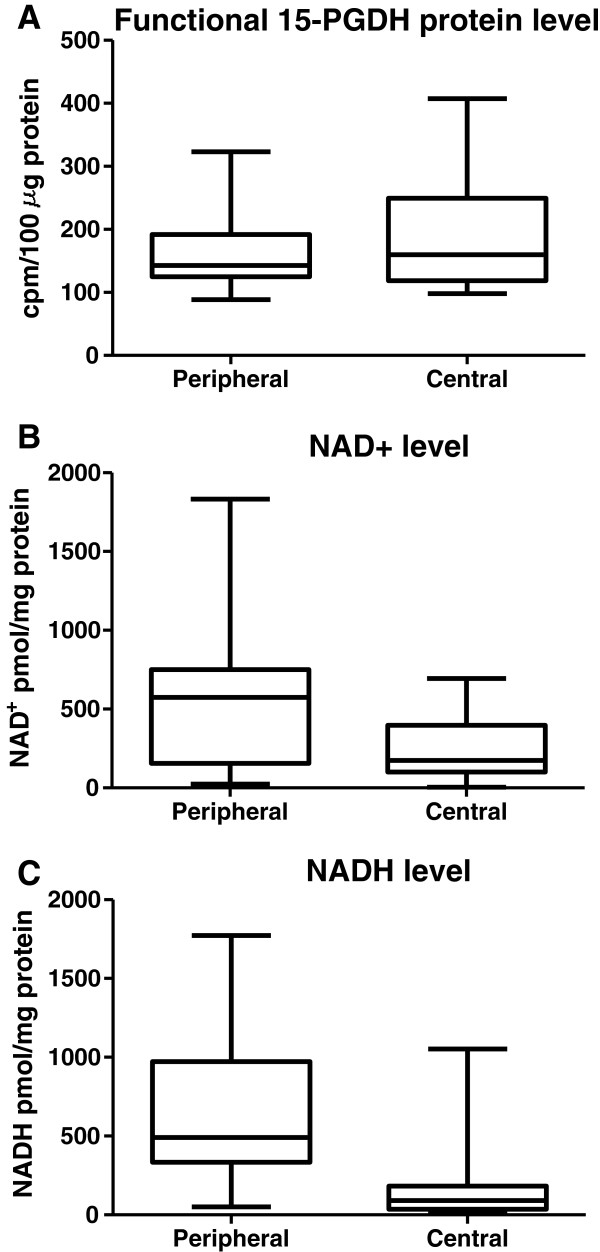
**Functional 15-PGDH protein levels are increased in the central area of CRCLM, but NAD+/NADH levels are decreased, relative to peripheral tumour tissue. A**) Maximal *ex vivo* 15-PGDH activity in CRCLM tissue in the presence of excess substrate and co-factors, **B**) NAD+ levels and **C**) NADH levels in peripheral and central CRCLM tissue. In each case, a box and whisker plot demonstrates the median, inter-quartile range and range of values.

The regional difference in intra-tumoral 15-PGDH immunoreactivity was confirmed by measurement of functional 15-PGDH protein levels by the 15-PGDH activity assay in the presence of excess substrate and co-factors. There was a median activity value of 160 cpm/100 μg protein (range 98–407) in central tumour regions and 142 cpm/100 μg protein (range 88–323 cpm/100 μg protein) in peripheral tumour regions (Figure 3A). 15-PGDH enzyme activity was higher in the central region of the tumour relative to the periphery in 14 of 20 CRC liver metastases (Additional file 5: Figure S4). 15-PGDH activity was 16% (95% CI: 3% – 29%) higher in the central tumour area compared with peripheral tumour tissue (p = 0.02).

Given the counter-intuitive observation that PGE_2_ levels were higher in the central area of CRCLM, in which expression of the main catabolic enzyme 15-PGDH was elevated, we performed a series of experiments, which were designed to investigate the relationship between 15-PGDH expression and levels of PGE_2_ in cell-conditioned medium, using HCA-7 human CRC cells, which constitutively express high levels of COX-2 and release large quantities of PGE_2_ into cell culture medium [28]. By contrast with the CRCLM tissue studies, we observed that reversible induction of 15-PGDH expression by acute exposure (less than 24 hours) to hypoxia (1% O_2_) was associated with a parallel reversible decrease in PGE_2_ levels in HCA-7 cell-conditioned medium, as expected (Additional file 6: Figure S5).

One explanation for high PGE_2_ levels in the presence of increased 15-PGDH protein expression in CRCLM, combined with the contrasting *in vitro* findings, is that 15-PGDH activity could be compromised by limiting amounts of NAD+ in a chronic hypoxic tumour microenvironment, with acute induction of 15-PGDH in HCA-7 human CRC cells being associated with a reduction in overall PGE_2_ production, possibly because there are sufficient cellular NAD+ stores to maintain efficient PGE_2_ catabolism in the acute setting. Therefore, we next addressed the hypothesis that NAD+/NADH levels are reduced in the central region of human CRCLM.

### NAD+ and NADH levels are lower in the central region of CRCLM relative to peripheral tumour tissue

The median NAD+ level in central tumour regions was 174 pmol/mg protein (range 5–695 pmol/mg protein) and 575 pmol/mg protein (range 24–1872 pmol/mg protein) in the peripheral CRCLM tissue (Figure 3B). We found that NAD+ levels were significantly lower in the central tumour area relative to peripheral tissue in 18 of 20 tumours (Additional file 5: Figure S4). There was a mean 59% (95% CI 25%-72%) reduction in NAD+ content in the tumour centre relative to peripheral tissue in paired CRCLM tissue (p = 0.01).

The median NADH level in central tumour regions was 90 pmol/mg protein (range 25–1053 pmol/mg protein) and 490 pmol/mg protein (range 50–1772 pmol/mg protein) in the peripheral tumour regions (Figure 3C). NADH levels were significantly lower in the central tumour area relative to peripheral tissue in 18 of 20 tumours (Additional file 5: Figure S4). There was a mean 76% (95% CI 61%-83%) reduction in NADH in the tumour centre relative to the peripheral area of the CRCLM (p < 0.01).

Paired data for NAD+ and NADH in central and peripheral tumour tissue were available for 15 CRCLMs. There was a higher NAD+/NADH ratio in the centre of the tumour (mean 3.01) compared with the CRCLM periphery (mean 1.29) in 9 of the 15 tumours but the median absolute difference in NAD+/NADH ratio between the centre and the periphery of CRCLMs was not statistically significant (1.63 [95% CI −0.69-3.66]).

### 15-PGDH enzyme activity is lower in hypoxic cancer cells relative to normoxic cancer cells

MCF-7 human breast cancer cells are known to have significant 15-PGDH activity [35] and hence were used as a model cancer cell system for initial experiments exploring the relationship between NAD+ availability and 15-PGDH activity. Using the 15-PGDH activity assay, we demonstrated that functional 15-PGDH protein expression was higher in cells cultured in hypoxia than normoxic conditions (913 vs. 763 cpm/mg protein), but the difference just failed to reach statistical significance (p = 0.07). This is consistent with the CRCLM data on 15-PGDH expression in the central region of CRCLMs and prompted the measurement of the effect of hypoxia on cellular NAD+ and NADH levels.

**Figure 4:**
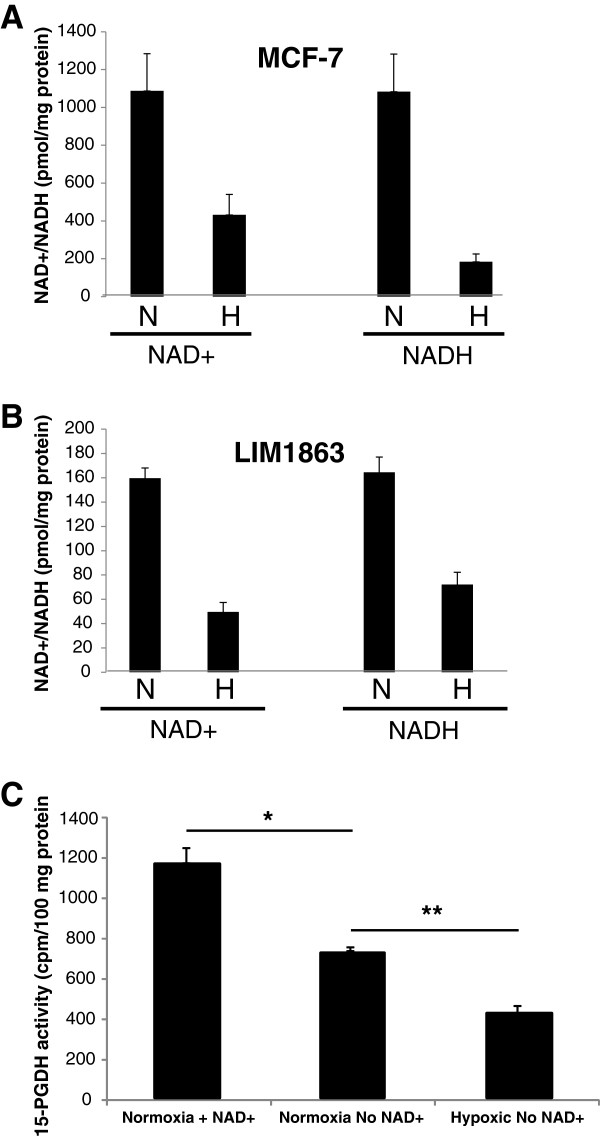
**The effect of hypoxia on NAD+/NADH levels and endogenous 15-PGDH activity in human cancer cells. A**) NAD+ and NADH levels in MCF-7 cells cultured in normoxia (20% O_2_; N) or hypoxia (1% O_2_; H) for 72 hours. Columns and bars represent the mean and the standard error of the mean (n = 5). **B**) NAD+ and NADH levels in LIM1863 cells cultured in normoxia (N) or hypoxia (H) for 72 hours. Columns and bars represent the mean and the standard error of the mean (n = 5). **C**) *Ex vivo* 15-PGDH activity assay of MCF-7 cell lysates in the presence or absence of exogenous NAD+ in the reaction mix. Columns and bars represent the mean and the standard error of the mean (n = 6).*p < 0.01, **p < 0.01.

In normoxic MCF-7 cells, median NAD+ and NADH levels were 1087 pmol/mg protein and 1084 pmol/mg protein respectively compared with median NAD+ and NADH values of 432 pmol/mg protein and 184 pmol/mg protein respectively in hypoxic MCF-7 cells (Figure 4A). A similar reduction was also seen in LIM 1863 human CRC cells, in which cells cultured in normoxia for 72 hours had median NAD+ and NADH levels of 160 pmol/mg protein and 164 pmol/mg protein respectively compared with median values of 50 pmol/mg protein and 72 pmol/mg protein in cells cultured in a hypoxic environmrnt for 72 hours (Figure 4B).

Since 15-PGDH is an NAD+ −dependent enzyme and NAD+ levels are significantly reduced in central tumour regions and hypoxic tumour cells, inefficient 15-PGDH enzyme function due to NAD+ depletion in hypoxia may explain the paradoxical finding of increased PGE_2_ levels in central regions of CRCLM in the presence of higher 15-PGDH protein levels.

We therefore tested whether low NAD+ levels in hypoxic cancer cells restricted 15-PGDH activity by measuring *ex vivo* 15-PGDH activity in MCF-7 cells in the presence and absence of exogenously added NAD+. Omission of exogenous NAD+ from the reaction mixture (so that 15-PGDH activity is dependent only on the presence of endogenous NAD+) when testing the lysate from cells cultured in normoxic conditions was associated with a significant reduction in 15-PGDH enzyme activity compared with cells supplemented with exogenous NAD+ (Figure 4C). Endogenous 15-PGDH activity in hypoxic MCF-7 cells was significantly lower than 15-PGDH activity in normoxic cells in the absence of exogenous NAD+ (Figure 4C), thus providing evidence that NAD+ levels may control 15-PGDH activity and hence affect PGE_2_ levels depending on the cellular oxygen tension.

### PGE_2_ promotes EMT in LIM 1863 human CRC cells

It has been described that PGE_2_ drives EMT of human CRC cells *in vitro*[36]. Therefore, we tested the effect of PGE_2_ on EMT of COX-2-positive LIM1863 human CRC cells, which can be used as an *in vitro* model of EMT in CRC [37,38]. LIM1863 cells exist in suspension under standard culture conditions. Upon treatment with recombinant human (rh) TGFβ, LIM1863 cells adhere to tissue culture plastic and grow as distinct colonies of cells, which have a mesenchymal phenotype (elongated, spindle-like cells with reduced E-cadherin expression) at the edge of the colony (Additional file 4: Figure S3). We used LIM1863 cell colony size following TGFβ treatment as an objective measure of EMT (Additional file 4: Figure S3). LIM1863 cells also have the advantage that they, like many human CRC cell lines, do not synthesize detectable quantities of PGE_2_ (data not shown), thereby allowing us to easily manipulate cell exposure to PGE_2_.

**Figure 5:**
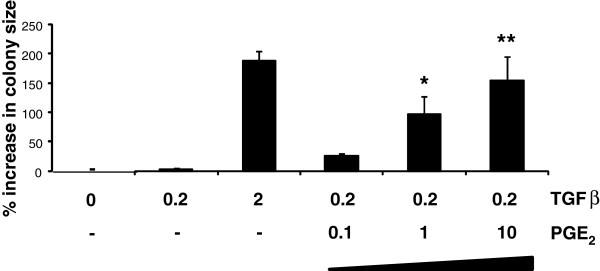
**PGE**_**2 **_**increases TGFβ-induced EMT in LIM1863 human CRC cells.** TGFβ induces EMT in LIM1863 human CRC cells in a concentration-dependent manner. Low concentration TGFβ (0.2 ng/ml) promotes LIM1863 human CRC cell colony adherence, but not spreading, unlike 2 ng/ml TGFβ, which induces an expansion in adherent colony size. PGE_2_ drives EMT in a concentration-dependent manner in LIM1863 cells exposed to low concentration TGFβ (0.2 ng/ml). Columns and bars demonstrate the mean and the standard error of the mean (n = 3).

Using our colony size assay, we confirmed previous data [39] that EMT in LIM1863 cells is induced by TGFβ in a concentration-dependent manner (Figure 5). Exogenous PGE_2_ (0.1-10 μM), in the presence of low concentration (0.2 ng/ml) rhTGFβ that induced LIM1863 cell colony adherence but minimal colony spreading, promoted EMT in LIM1863 cells in a concentration-dependent manner (Figure 5). However, PGE_2_ did not induce EMT of LIM1863 human CRC cells in the absence of TGFβ (data not shown).

### Hypoxia promotes EMT in LIM 1863 cells

**Figure 6:**
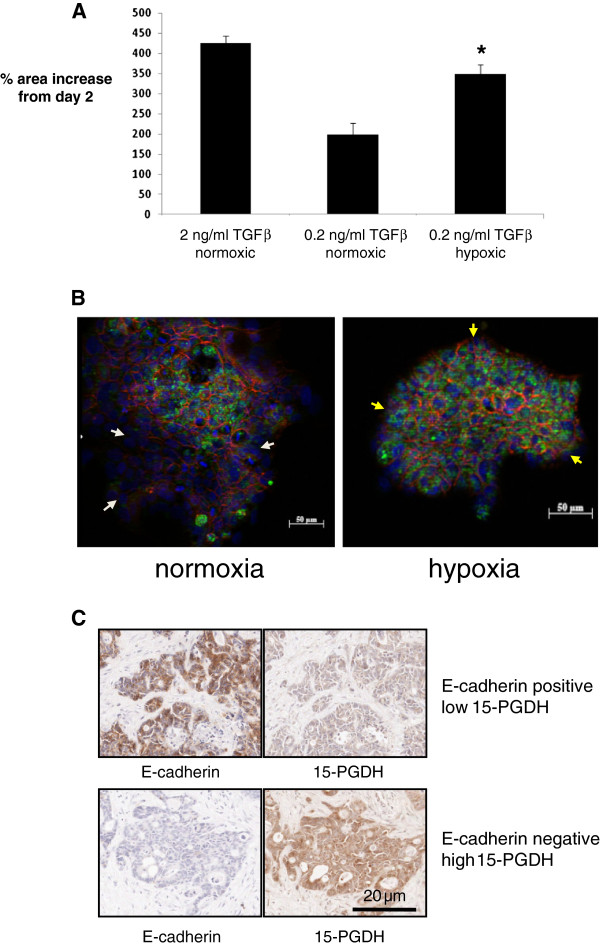
**Hypoxia promotes EMT of LIM1863 human CRC cells. A**) EMT of LIM1863 human CRC cells cultured in either normoxia (20% O_2_) or hypoxia (1% O_2_) in the presence of 0.2 ng/ml TGFβ. Positive control cells underwent EMT in the presence of 2 ng/ml TGFβ. *p = 0.02 for the difference from normoxic cells cultured in the presence of 0.2 ng/ml TGFβ (n = 3). **B**) Dual immunofluorescence for E-cadherin (red) and 15-PGDH (green) on LIM1863 human CRC cells six days after addition of TGFβ. DAPI was used for nuclear visualisation (blue). LIM 1863 human CRC cells were cultured in the presence of 0.2 ng/ml TGFβ for six days (normoxia) or were transferred to hypoxic conditions (1% O2) on day two. In normoxic conditions, E-cadherin-negative cells at the edge of the colony did not express 15-PGDH (white arrows). However, E-cadherin-low cells at the edge of hypoxic colonies were still 15-PGDH-positive (yellow arrows). **C**) Immunohistochemistry for 15-PGDH and E-cadherin on sequential sections of a representative central region of CRCLM. Images taken from representative areas of stained sections show an inverse relationship between E-cadherin and 15-PGDH immunoreactivity such that 15-PGDH expression was high in E-cadherin-low cells and vice versa.

As we had previously demonstrated that hypoxia limits 15-PGDH activity and is associated with increased PGE_2_ levels in the central region of CRCLMs, we then tested whether hypoxia promoted EMT and affected 15-PGDH expression in LIM1863 cells. Hypoxia significantly promoted EMT of LIM1863 cells compared with normoxic conditions (Figure 6A). In LIM1863 cell colonies cultured in normoxia, cells at the edge of the colony exhibited reduced membranous E-cadherin expression (Figure 6B), in keeping with a mesenchymal phenotype as described [37]. These cells contained less 15-PGDH than cells in the centre of the colony (Figure 6B). By contrast, hypoxic LIM 1863 cell colonies did not display any reduction in 15-PGDH protein content in cells at the edge of the colony compared with cells in the centre of a colony (Figure 6B). Observations consistent with these *in vitro* findings were made in human CRCLM tissue, in which there was an inverse relationship between 15-PGDH and E-cadherin immunoreactivity in tumour cells in central areas of CRCLMs (Figure 6C). In particular, CRC cells that had lost E-cadherin expression contained higher levels of immunoreactive 15-PGDH protein consistent with the observations on hypoxic LIM1863 cells Figure 6C). By contrast, this relationship was not observed in CRC cells in the periphery of CRCLMs, in which E-cadherin-low cells had lower 15-PGDH protein expression than cells that maintained membranous E-cadherin expression (data not shown).

## Discussion

This is the first study to report regional differences in the levels of PGE_2_ and 15-PGDH in human colorectal tumours. This was made possible by employing a strict protocol for rapid and uniform processing of orientated tumour tissue *ex vivo*. Herein, we report that PGE_2_ levels are higher towards the centre of CRCLM compared with more peripheral cancer tissue. Paradoxically, this was associated with increased levels of 15-PGDH *protein* at the centre of CRCLM. However, we demonstrated that the 15-PGDH *activity* level in the centre of CRCLM is reduced and is associated with low NAD+/NADH levels. *In vitro* studies confirmed that NAD+ availability drives 15-PGDH activity in human CRC cells. We believe that consideration of regional differences in PGE_2_ metabolism and micro-environmental influences on PGE_2_ metabolism related to enzyme co-factor availability and/or hypoxia is a paradigm shift in the field of eicosanoid cancer research and is consistent with latest understanding of genetic and epigenetic intra-tumoral heterogeneity [14,40]. Consideration of intra-tumoral differences in PGE_2_ metabolism is essential for development of optimal anti-CRC therapy aimed at the COX-PGE_2_-15-PGDH axis.

Our data highlight significant differences between findings in human cancer tissue *ex vivo* and experimental observations using CRC cells *in vitro*. Although we propose that differences in 15-PGDH *activity* in cancer tissue compared with cultured CRC cells may account for the contrasting relationship between 15-PGDH expression and PGE_2_ levels in CRCLM tissue versus cell-conditioned medium, we cannot completely rule out that inadvertent stimulation of PGE_2_ synthesis *ex vivo* occurred. Avoidance of possible artefactual changes in tissue eicosanoid levels *ex vivo* will only be possible with other techniques such as MALDI-MS for measurement of PG distribution in frozen tissue sections [41].

The tissue microarray comparison of regional differences in 15-PGDH immunoreactivity between CRCLM and the paired primary CRC suggests that 15-PGDH expression, and hence PGE_2_ metabolism, in CRCLM differs from that in the primary CRC, from which the CRCLM were derived. This finding is consistent with recent data describing significant genetic differences between primary CRC and synchronous liver metastasis [40]. Local factors specific to CRCLM may, at least partly, explain regional 15-PGDH expression in CRCLM and the contrast with observations from previous studies of 15-PGDH expression in primary CRCs [12].

NAD+ and NADH levels were both significantly lower in central rather than peripheral CRCLM tissue, compatible with depletion of the cellular NAD(H) pool. The NAD+/NADH ratios that we observed in human CRCLM tissue are similar to previous studies that have measured tissue NAD(H) levels by the same cycling assay [42]. However, absolute levels of NAD+ and NADH were low compared with other tissues [42]. One testable hypothesis is that the NAD(H) pool is depleted because of increased NAD-consuming enzyme activity in CRC cells. Consistent with this notion, sirtuins such as SIRT1 and poly-(ADP ribose) polymerase expression and activity are increased in cancer tissue [43]. In particular, SIRT1 expression and activity are increased in human hepatoma and fibrosarcoma cells *in vitro*[26].

One weakness of our study is that we do not have direct evidence that the central area of CRCLMs that we studied were hypoxic. However, there is substantial indirect evidence that regional hypoxia exists in tumours including CRCLMs [19-21]. Importantly, the regional difference in functional 15-PGDH protein levels in CRCLMs was not mirrored in primary CRC. Central tumour necrosis is more common in CRCLMs than primary CRC tumours and implies greater degrees of hypoxia in the central regions of CRCLMs, which could account for differential 15-PGDH expression in metastatic tumours. This observation, and the fact that elevated 15-PGDH in CRC cells in the centre of CRCLMs is likely inactive secondary to NAD+ deficiency, help to reconcile our data with the existing literature, which, in general (but not exclusively), implies that 15-PGDH has tumour suppressor activity [12].

Roberts et al. have reported that acute hypoxia (16 hours) did not alter 15-PGDH protein expression in HT-29 human CRC cells, despite an increase in PGE_2_ levels believed to be secondary to COX-2 induction [25]. It is possible that CRC cell line-specific differences in hypoxia-induced gene expression and NAD+ availability explain the experimental variability in *in vitro* models. Nevertheless, our data highlight that it is crucial to confirm the relevance of *in vitro* observations in tissue expression studies, which take into account potential micro-environmental influences.

TGFβ-induced attachment and spreading of LIM1863 human CRC cell colonies allowed us to develop a novel semi-quantitative measure of EMT based on an established model [38]. Using this assay, we have provided support for previous observations that PGE_2_ drives EMT of CRC and other human cancer cells *in vitro*, which were based on down-regulation of E-cadherin expression, light-microscopic phenotype changes in adherent cells and cell motility assays [5,36,44,45].

We have contributed to emerging evidence that hypoxia drives EMT [46]. Interestingly, we observed that 15-PGDH expression was maintained in hypoxic TGFβ-induced LIM1863 human CRC cell colonies *in vitro* and CRC cells in the centre of CRCLMs that had an ‘EMT (E-cadherin-low) phenotype’. This is consistent with our observations that hypoxia induces 15-PGDH in other CRC cell lines *in vitro* and that 15-PGDH levels are higher in the centre rather than the periphery of CRCLMs. One testable hypothesis is that hypoxia inhibits β-catenin-related signaling [47], which could lead to de-repression of 15-PGDH [48]. Further studies are required to understand the rather counter-intuitive finding that the main rate-limiting catabolic enzyme for PGE_2_ inactivation is elevated in a tumour microenvironment, in which cell survival would be potentiated by PGE_2_. These studies should always take into account NAD+ co-factor availability and measure levels of other lipid mediators, which have anti-proliferative activity, that are also potential substrates for 15-PGDH such as lipoxins [11].

Previous *in vitro* studies have demonstrated that Snail, one of the key transcription factors in EMT, represses 15-PGDH expression in CRC cells via direct binding to conserved E-box elements in the 15-PGDH promoter region [5]. However, to our knowledge, the effect of hypoxia on human 15-PGDH gene expression has not been formally assessed. The human 15-PGDH gene promoter contains multiple ETS, AP-1 and CREB binding sites [49], although no hypoxia-responsive elements for direct hypoxia-inducible factor binding are evident. Therefore, a valid, testable hypothesis is that 15-PGDH is a hypoxia-inducible gene in CRC via ETS-dependent transcriptional up-regulation, which is recognised for several hypoxia-inducible genes [50].

## Conclusions

In summary, we have demonstrated that there are significant regional differences in PGE_2_ metabolism in human CRCLM. Relative lack of NAD+ availability in the central tumour micro-environment is a plausible explanation for the difference in relationship between PGE_2_ content and 15-PGDH expression in tumour tissue compared with cultured human CRC cells *in vitro*. A reciprocal relationship between the PGE_2_ level and 15-PGDH expression is lost in hypoxic conditions in the context of CRC EMT. It will be essential to measure 15-PGDH function rather than just protein levels in future studies of PGE_2_ availability and the role of 15-PGDH during human carcinogenesis.

## Competing interest

The authors declare that they have no competing interests.

## Authors’ contributions

ALY collected all tissue samples and performed *in vitro* studies; CRC and GH performed CRC cell experiments; SLP performed immunohistochemistry; DT supervised the immunohistochemical scoring; GJT, PJ and MAH conceived of the study, and participated in its design and coordination and helped to draft the manuscript. All authors read and approved the final manuscript.

## Pre-publication history

The pre-publication history for this paper can be accessed here:

http://www.biomedcentral.com/1471-2407/13/92/prepub

## Supplementary Material

Additional file 1 Methods. Click here for file

Additional file 2: Figure S1 Quantitiative immunohistochemistry analysis. Click here for file

Additional file 3: Figure S2 15-PGDH IHC on tissue microarrays of primary CRCs and matched CRCLM. Click here for file

Additional file 4: Figure S3 LIM1863 human CRC cells cultured in the absence. Click here for file

Additional file 5: Figure S4 Individual paired values of enzyme immunoreactivity, activity and NAD/NADH levels from the periphery (blue) and centre (yellow) of 20 CRCLM. Click here for file

Additional file 6: Figure S5 The effect of acute hypoxia 15-PGDH mRnA expression and PGE_2_ levels in medium conditioned by HCA-7 human cRc cells. Click here for file
