# Supplementary material for: Regional differences in prostaglandin E2 metabolism in human colorectal cancer liver metastases
Source: BMC Cancer. 2013 Feb 26;13:92. doi: 10.1186/1471-2407-13-92 (PMC3598740; doi:10.1186/1471-2407-13-92)
Supplement: Additional file 1 — Methods. [file 1471-2407-13-92-S1.docx]

**Supplementary Methods**

**PGE_2_ assay**

Tumour tissue for PGE_2_ measurement was immediately placed in proprietary lysis buffer (Amersham Biosciences, Amesham, UK), incorporating 1 µM indomethacin (Sigma, Poole, UK) in order to prevent PGE_2_ production *ex vivo*. Tissue was homogenized using a Wheaton dounce tissue grinder. The homogenate was cleared by centrifugation at 1000 *g* for 5 minutes. The number of viable (trypan blue-negative) human CRC cells was counted using a haemocytometer for calculation of PGE_2_ levels in cell-conditioned medium.

**Immunohistochemistry**

Sections for 15-PGDH and E-cadherin IHC were deparaffinised and rehydrated in xylene and ethanol series respectively and then endogenous peroxidase activity was blocked with hydrogen peroxide (0.3% (v/v) 15-PGDH; 3% (v/v) E-cadherin) in 100% methanol for 10 minutes. Antigen retrieval was by pressure cooker for 15-PGDH and microwave heating for E-cadherin (2 minutes each), in 10 mM citrate buffer, pH 6.0. For 15-PGDH IHC, biotin and non-specific protein binding blocks (all Vector Laboratories) were performed prior to incubation with a rabbit polyclonal antibody to 15-PGDH (Cayman Chemical Co., Ann Arbor, MI) at a dilution of 1:200 for 1 hour at 25°C before being washed with phosphate-buffered saline (PBS) and application of a swine anti-rabbit biotinylated secondary antibody (DakoCytomation, Ely, UK) at a 1:150 dilution for 1 hour at 25°C. Avidin-biotin-peroxidase complex (Vector) was applied for 30 minutes before diaminobenzidine (DAB) (0.02%) was applied for 5 minutes to produce a chromogenic product. For E-cadherin, antibody diluent (Invitogen) was applied to the slide to block non-specific binding before mouse monoclonal antibody to E-cadherin (Invitrogen) was applied for 1 hour at 25°C at a 1:50 dilution in antibody diluent. This was then washed with Tris-buffered saline and horseradish peroxidase-conjugated anti-mouse antibody (Invitrogen) at 1:150 in antibody diluent was applied for 30 minutes before DAB exposure as above.

**Quantitative immunohistochemistry analysis**

See Supplementary Figure 1. Slides were scanned at x40 magnification using a Scanscope XT (Aperio, Vista, CA, USA) and then analysed using Imagescope (Aperio v8.2) software. A horizontal line was drawn along the length of the tumour-liver border of each section. A perpendicular line was then drawn from the mid-point of the tumour-liver line to the central border of the section. A 1 mm portion of the perpendicular line above tumour tissue was excluded to avoid any edge staining artefact. The perpendicular line was then divided into three equal lengths. A rectangle was created 150 µm wide and one third of the tumour section in length adjacent to the outer and inner thirds of the perpendicular line, which represented peripheral and central tumour regions for analysis respectively. Within each box, all CRC epithelial cells were highlighted (excluding stroma and necrosis) in order to analyze the intensity of staining of every pixel within the tumour cell region of interest using the computer software. A minimum of 10 000 pixels were measured in each region.

**15-PGDH enzyme activity assay**

15-PGDH enzyme activity in CRCLM tissue was measured by assessing the transfer of tritium from [^3^H]PGE_2_ to glutamate, catalysed by 15-PGDH and glutamate dehydrogenase (Tai, *Biochemistry* 1976;15:4586-92). Tumour cell lysate was produced by homogenization of tissue by mechanical grinding with a plastic plunger or pipetting of cells, followed by centrifugation in a QIAshredder (QIAGEN). The reaction mix contained potassium phosphate buffer (pH 7.5), 1 µmol α-ketoglutarate, 5 µmol ammonium chloride, 100 µg glutamate dehydrogenase (Sigma), 1 nM [^3^H]-PGE_2_  and 1 µmol NAD+ (Sigma) in a final volume of 1 ml. The reaction proceeded at 37°C for 10 minutes, at which point it was terminated by the addition of 0.3 ml of 10% (w/v) activated charcoal solution with 1% dextran (w/v) in order to remove excess [^3^H]-PGE_2_. Following centrifugation (1000 *g* for 5 minutes), 500 µl supernatant was aspirated and analysed in a 1450 MicroBeta Jet scintillation counter (Zeiss). Data are expressed as cpm per 100 mg/protein. Any values below the negative control (MCF-7 cell lysate heat-inactivated at 101°C for 15 minutes) were excluded.

**Immunofluorescence**

LIM1863 cells were cultured in plastic cell culture dishes (Zeiss) before being fixed in methanol at -20°C for 20 minutes. A 10% (w/v) skimmed milk solution in PBS was used as a blocking agent and antibody diluent. Following a PBS wash, donkey anti-rabbit, Alexa Fluor® 488 (for 15-PGDH) and goat anti-mouse Alexa Fluor® 594 (for E-cadherin; both Invitrogen) were applied for one hour at 25°C at a dilution of 1:300. PBS was used to wash off the secondary antibody before application of Prolong Gold™ mountant containing 4',6-diamidino-2-phenylindole (DAPI).
