# Supplementary material for: Regional differences in prostaglandin E2 metabolism in human colorectal cancer liver metastases
Source: BMC Cancer. 2013 Feb 26;13:92. doi: 10.1186/1471-2407-13-92 (PMC3598740; doi:10.1186/1471-2407-13-92)
Supplement: Additional file 2: Figure S1 — Quantitiative immunohistochemistry analysis. [file 1471-2407-13-92-S2.pptx]

## Slide 1
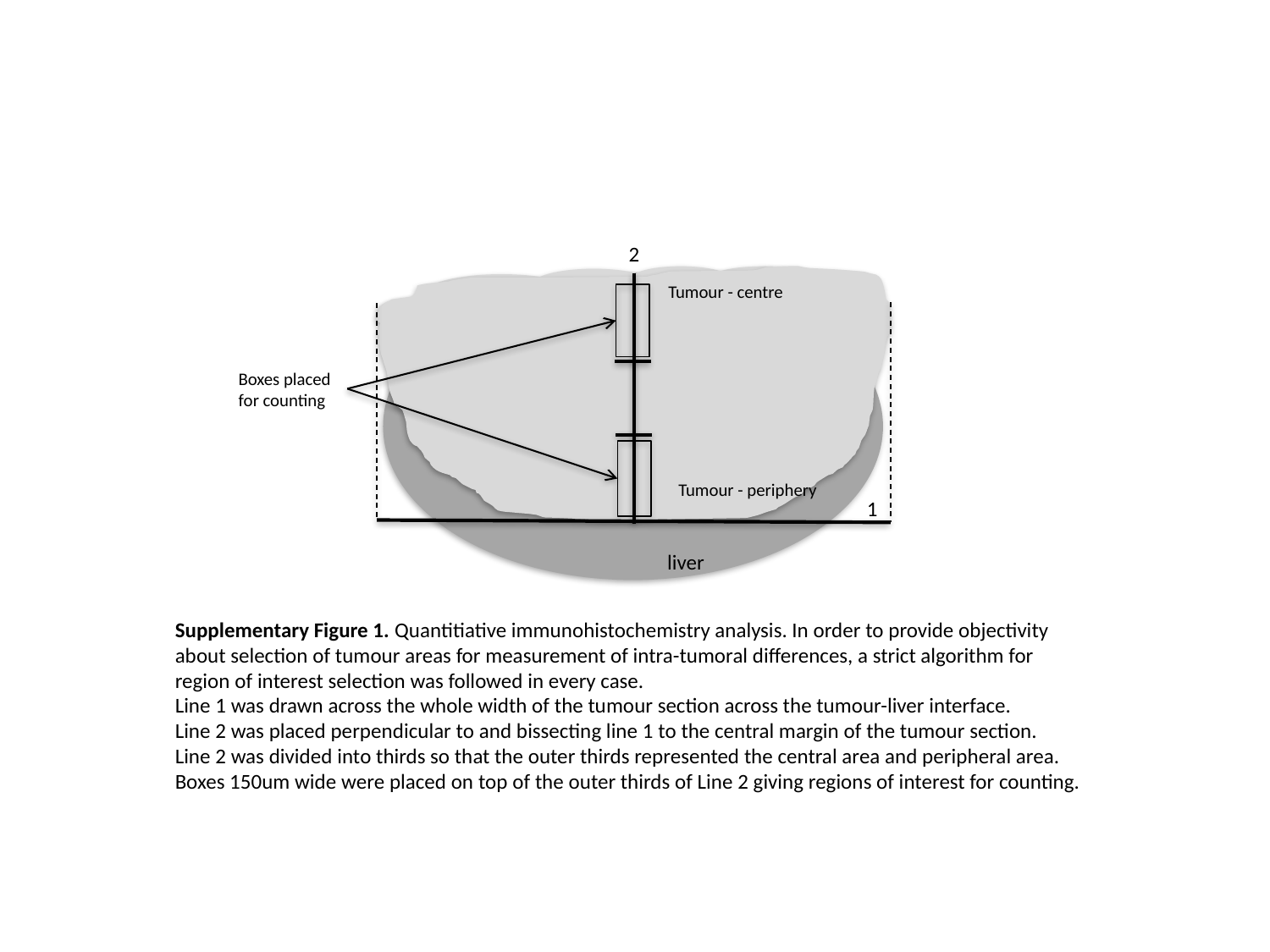

2
Tumour - centre
Boxes placed
for counting
Tumour - periphery
1
liver
Supplementary Figure 1. Quantitiative immunohistochemistry analysis. In order to provide objectivity about selection of tumour areas for measurement of intra-tumoral differences, a strict algorithm for region of interest selection was followed in every case.
Line 1 was drawn across the whole width of the tumour section across the tumour-liver interface.
Line 2 was placed perpendicular to and bissecting line 1 to the central margin of the tumour section.
Line 2 was divided into thirds so that the outer thirds represented the central area and peripheral area.
Boxes 150um wide were placed on top of the outer thirds of Line 2 giving regions of interest for counting.
