# Supplementary material for: Regional differences in prostaglandin E2 metabolism in human colorectal cancer liver metastases
Source: BMC Cancer. 2013 Feb 26;13:92. doi: 10.1186/1471-2407-13-92 (PMC3598740; doi:10.1186/1471-2407-13-92)
Supplement: Additional file 3: Figure S2 — 15-PGDH IHC on tissue microarrays of primary CRCs and matched CRCLM. [file 1471-2407-13-92-S3.pptx]

## Slide 1
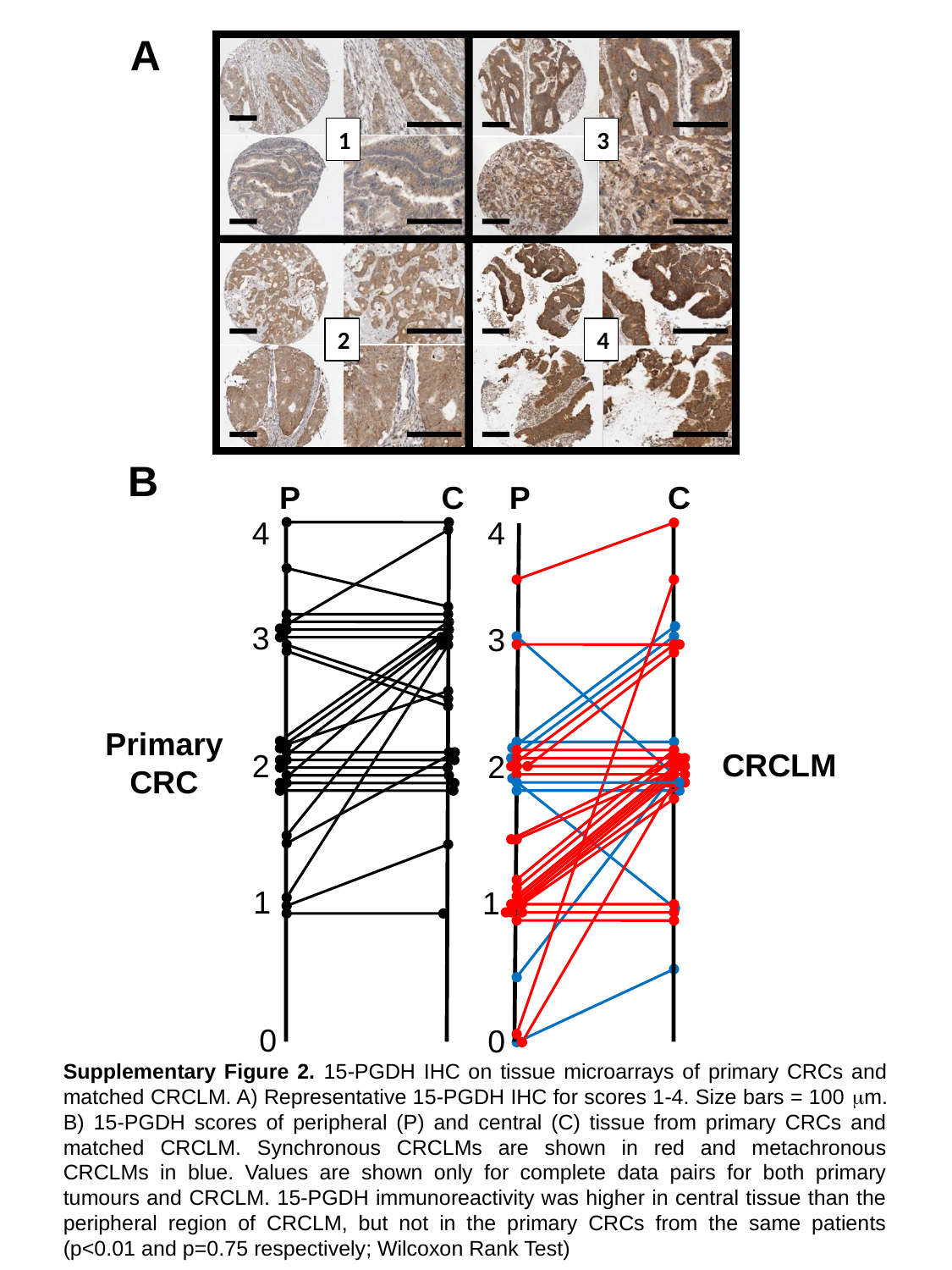

A
1
3
2
2
4
B
 P
 C
 P
 C
4
4
3
3
Primary CRC
CRCLM
2
2
1
1
0
0
Supplementary Figure 2. 15-PGDH IHC on tissue microarrays of primary CRCs and matched CRCLM. A) Representative 15-PGDH IHC for scores 1-4. Size bars = 100 mm. B) 15-PGDH scores of peripheral (P) and central (C) tissue from primary CRCs and matched CRCLM. Synchronous CRCLMs are shown in red and metachronous CRCLMs in blue. Values are shown only for complete data pairs for both primary tumours and CRCLM. 15-PGDH immunoreactivity was higher in central tissue than the peripheral region of CRCLM, but not in the primary CRCs from the same patients (p<0.01 and p=0.75 respectively; Wilcoxon Rank Test)
