# Supplementary material for: Regional differences in prostaglandin E2 metabolism in human colorectal cancer liver metastases
Source: BMC Cancer. 2013 Feb 26;13:92. doi: 10.1186/1471-2407-13-92 (PMC3598740; doi:10.1186/1471-2407-13-92)
Supplement: Additional file 4: Figure S3 — LIM1863 human CRC cells cultured in the absence. [file 1471-2407-13-92-S4.pptx]

## Slide 1
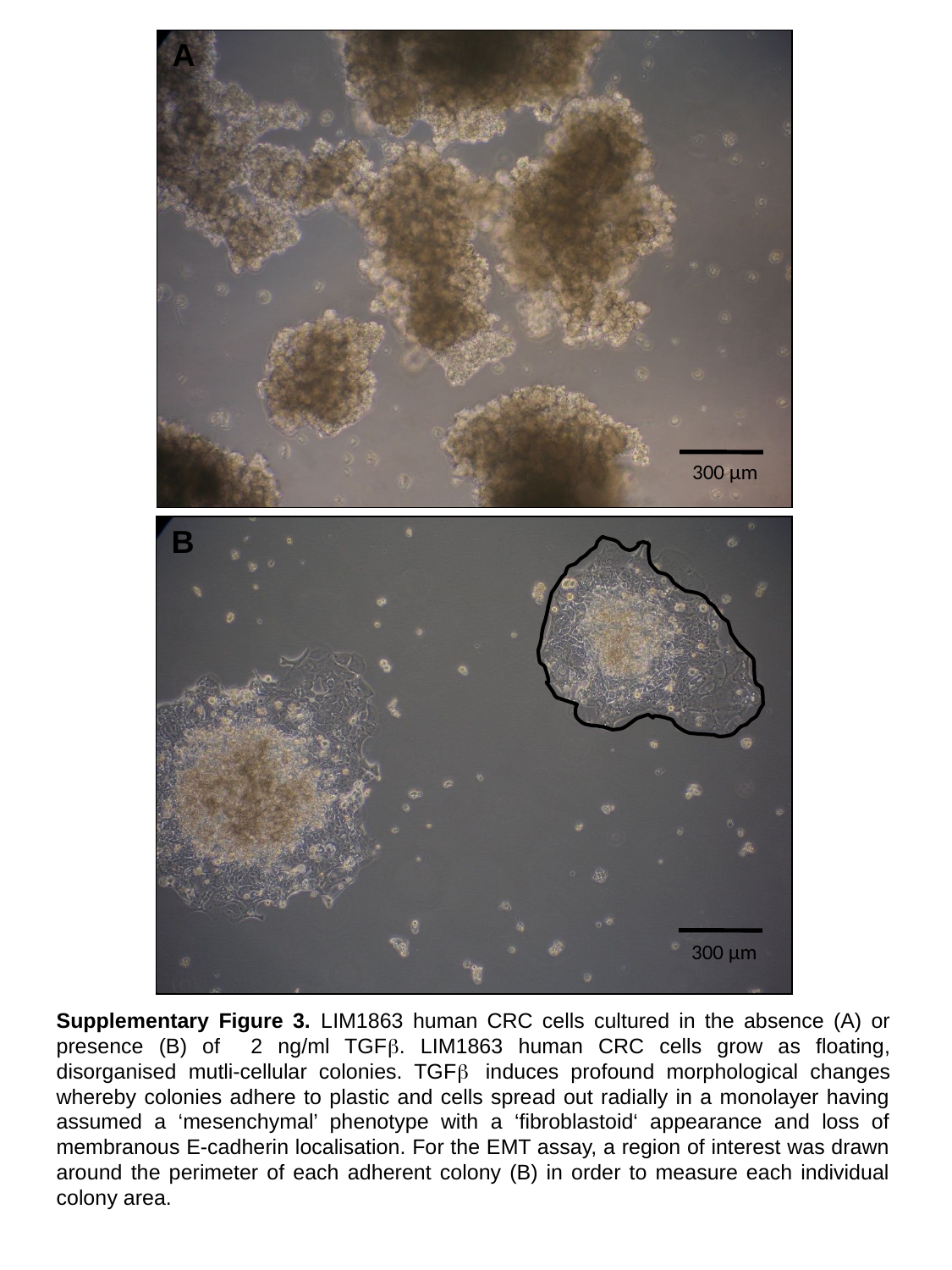

A
300 µm
B
300 µm
Supplementary Figure 3. LIM1863 human CRC cells cultured in the absence (A) or presence (B) of 2 ng/ml TGFb. LIM1863 human CRC cells grow as floating, disorganised mutli-cellular colonies. TGFb induces profound morphological changes whereby colonies adhere to plastic and cells spread out radially in a monolayer having assumed a ‘mesenchymal’ phenotype with a ‘fibroblastoid‘ appearance and loss of membranous E-cadherin localisation. For the EMT assay, a region of interest was drawn around the perimeter of each adherent colony (B) in order to measure each individual colony area.
