# Supplementary material for: Regional differences in prostaglandin E2 metabolism in human colorectal cancer liver metastases
Source: BMC Cancer. 2013 Feb 26;13:92. doi: 10.1186/1471-2407-13-92 (PMC3598740; doi:10.1186/1471-2407-13-92)
Supplement: Additional file 5: Figure S4 — Individual paired values of enzyme immunoreactivity, activity and NAD/NADH levels from the periphery (blue) and centre (yellow) of 20 CRCLM. [file 1471-2407-13-92-S5.pptx]

## Slide 1
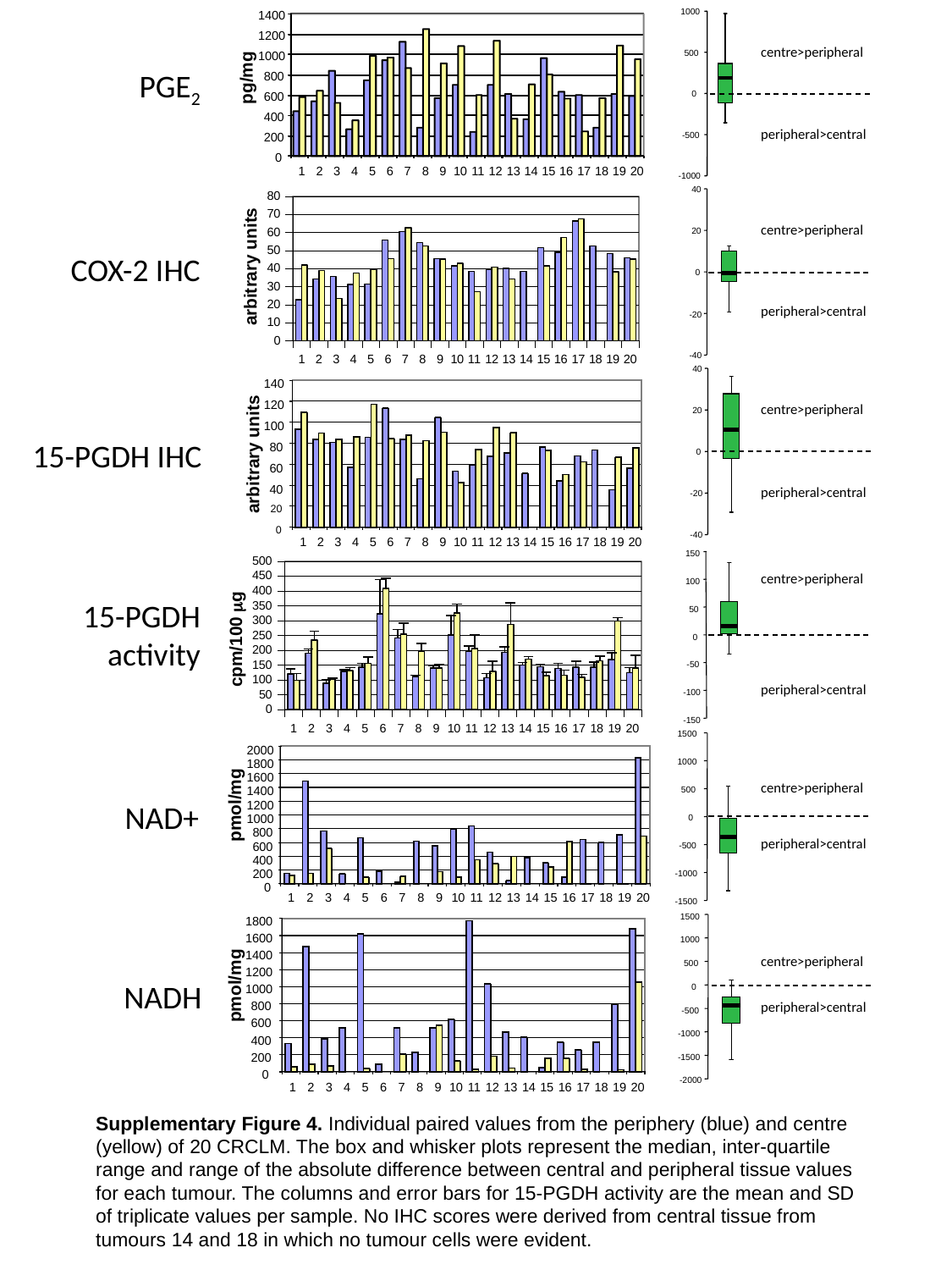

1000
500
0
-500
-1000
1400
1200
1000
800
600
400
200
0
1
2
3
4
5
6
7
8
9
10
11
12
13
14
15
16
17
18
19
20
PGE2
pg/mg
centre>peripheral
peripheral>central
### Chart
| Category | Periphery | Centre |
|---|---|---|
| 1 | 22.644000000000005 | 41.900000000000006 |
| 2 | 34.24200000000001 | 39.05699999999999 |
| 3 | 35.68000000000001 | 23.391999999999996 |
| 4 | 31.217999999999993 | 37.44200000000001 |
| 5 | 31.32400000000003 | 39.72800000000021 |
| 6 | 55.92800000000001 | 45.505 |
| 7 | 60.464000000000006 | 62.717000000000006 |
| 8 | 54.322 | 52.572 |
| 9 | 45.461000000000006 | 45.2250000000002 |
| 10 | 41.59300000000001 | 42.845 |
| 11 | 38.409000000000006 | 27.27000000000001 |
| 12 | 39.29100000000001 | 40.79400000000001 |
| 13 | 40.21600000000001 | 34.15799999999999 |
| 14 | 38.452999999999996 | 0.0 |
| 15 | 51.49200000000001 | 41.40799999999999 |
| 16 | 49.017 | 57.27900000000001 |
| 17 | 66.33600000000001 | 67.61699999999999 |
| 18 | 52.516000000000005 | 0.0 |
| 19 | 48.23800000000006 | 38.251000000000005 |
| 20 | 46.0 | 45.364000000000004 |40
20
0
-20
-40
COX-2 IHC
arbitrary units
centre>peripheral
peripheral>central
40
140
120
100
80
60
40
20
0
1
2
3
4
5
6
7
8
9
10
11
12
13
14
15
16
17
18
19
20
20
15-PGDH IHC
arbitrary units
0
-20
-40
centre>peripheral
peripheral>central
150
### Chart
| Category | Periphery | Centre |
|---|---|---|100
15-PGDH activity
50
cpm/100 mg
0
-50
-100
-150
centre>peripheral
peripheral>central
1500
2000
1800
1000
pmol/mg
1600
centre>peripheral
1400
500
NAD+
1200
1000
0
800
peripheral>central
600
-500
400
200
-1000
0
1
2
3
4
5
6
7
8
9
10
11
12
13
14
15
16
17
18
19
20
-1500
1500
1800
1600
1000
pmol/mg
centre>peripheral
1400
500
1200
NADH
0
1000
peripheral>central
800
-500
600
-1000
400
200
-1500
0
-2000
1
2
3
4
5
6
7
8
9
10
11
12
13
14
15
16
17
18
19
20
Supplementary Figure 4. Individual paired values from the periphery (blue) and centre (yellow) of 20 CRCLM. The box and whisker plots represent the median, inter-quartile range and range of the absolute difference between central and peripheral tissue values for each tumour. The columns and error bars for 15-PGDH activity are the mean and SD of triplicate values per sample. No IHC scores were derived from central tissue from tumours 14 and 18 in which no tumour cells were evident.
