# Supplementary material for: Regional differences in prostaglandin E2 metabolism in human colorectal cancer liver metastases
Source: BMC Cancer. 2013 Feb 26;13:92. doi: 10.1186/1471-2407-13-92 (PMC3598740; doi:10.1186/1471-2407-13-92)
Supplement: Additional file 6: Figure S5 — The effect of acute hypoxia 15-PGDH mRnA expression and PGE2 levels in medium conditioned by HCA-7 human cRc cells. [file 1471-2407-13-92-S6.pptx]

## Slide 1
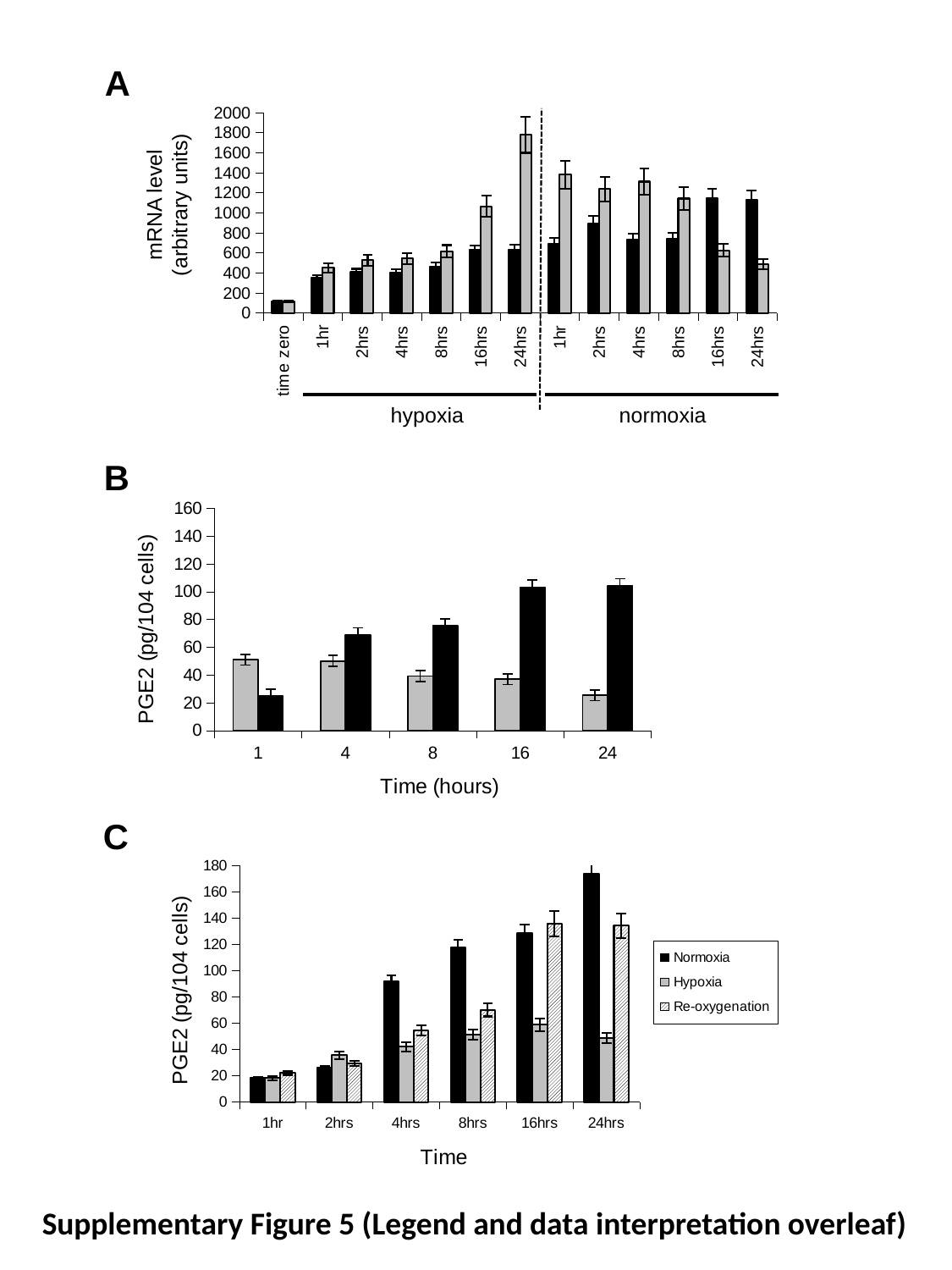

A
### Chart
| Category | Normoxia | Hypoxia |
|---|---|---|
| time zero | 116.0 | 116.0 |
| 1hr | 350.0 | 451.0 |
| 2hrs | 408.0 | 526.0 |
| 4hrs | 405.0 | 544.0 |
| 8hrs | 465.0 | 616.0 |
| 16hrs | 627.0 | 1065.0 |
| 24hrs | 630.0 | 1776.0 |
| 1hr | 693.0 | 1380.0 |
| 2hrs | 894.0 | 1236.0 |
| 4hrs | 730.0 | 1311.0 |
| 8hrs | 738.0 | 1142.0 |
| 16hrs | 1147.0 | 625.0 |
| 24hrs | 1132.0 | 489.0 |mRNA level
(arbitrary units)
hypoxia
normoxia
B
### Chart
| Category | Hypoxia | Normoxia |
|---|---|---|
| 1 | 50.956521739130395 | 24.9 |
| 4 | 49.9913043478261 | 68.88799999999999 |
| 8 | 39.134782608695645 | 75.46400000000003 |
| 16 | 36.891304347826086 | 103.33599999999998 |
| 24 | 25.31304347826087 | 104.316 |C
### Chart
| Category | Normoxia | Hypoxia | Re-oxygenation |
|---|---|---|---|
| 1hr | 18.14 | 18.09 | 22.19 |
| 2hrs | 26.05 | 35.54 | 29.23 |
| 4hrs | 92.0 | 41.949999999999996 | 54.46 |
| 8hrs | 117.31 | 51.26000000000001 | 69.97 |
| 16hrs | 128.62 | 58.75 | 135.67 |
| 24hrs | 173.68 | 48.36 | 134.2 |Supplementary Figure 5 (Legend and data interpretation overleaf)

## Slide 2
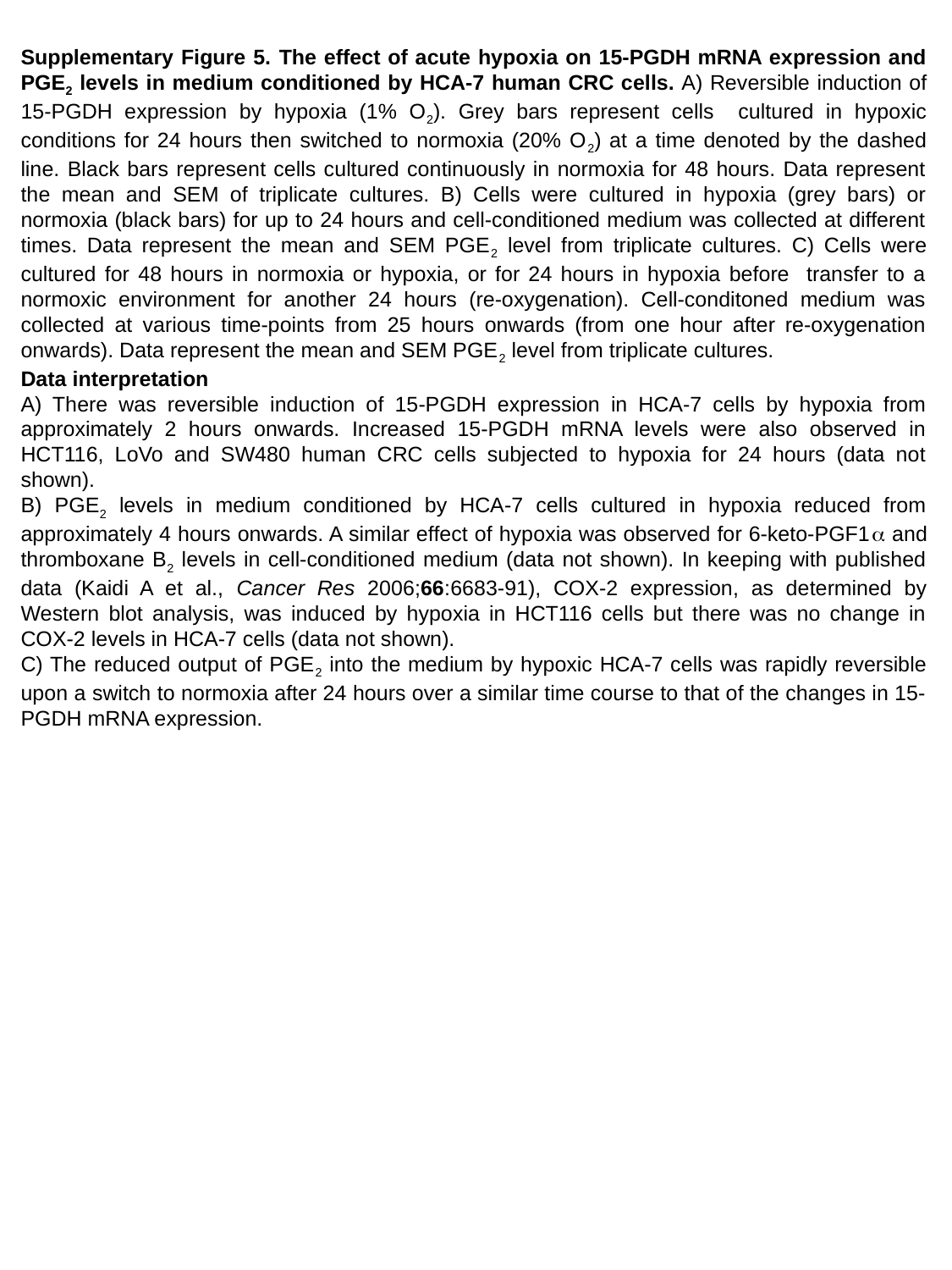

Supplementary Figure 5. The effect of acute hypoxia on 15-PGDH mRNA expression and PGE2 levels in medium conditioned by HCA-7 human CRC cells. A) Reversible induction of 15-PGDH expression by hypoxia (1% O2). Grey bars represent cells cultured in hypoxic conditions for 24 hours then switched to normoxia (20% O2) at a time denoted by the dashed line. Black bars represent cells cultured continuously in normoxia for 48 hours. Data represent the mean and SEM of triplicate cultures. B) Cells were cultured in hypoxia (grey bars) or normoxia (black bars) for up to 24 hours and cell-conditioned medium was collected at different times. Data represent the mean and SEM PGE2 level from triplicate cultures. C) Cells were cultured for 48 hours in normoxia or hypoxia, or for 24 hours in hypoxia before transfer to a normoxic environment for another 24 hours (re-oxygenation). Cell-conditoned medium was collected at various time-points from 25 hours onwards (from one hour after re-oxygenation onwards). Data represent the mean and SEM PGE2 level from triplicate cultures.
Data interpretation
A) There was reversible induction of 15-PGDH expression in HCA-7 cells by hypoxia from approximately 2 hours onwards. Increased 15-PGDH mRNA levels were also observed in HCT116, LoVo and SW480 human CRC cells subjected to hypoxia for 24 hours (data not shown).
B) PGE2 levels in medium conditioned by HCA-7 cells cultured in hypoxia reduced from approximately 4 hours onwards. A similar effect of hypoxia was observed for 6-keto-PGF1a and thromboxane B2 levels in cell-conditioned medium (data not shown). In keeping with published data (Kaidi A et al., Cancer Res 2006;66:6683-91), COX-2 expression, as determined by Western blot analysis, was induced by hypoxia in HCT116 cells but there was no change in COX-2 levels in HCA-7 cells (data not shown).
C) The reduced output of PGE2 into the medium by hypoxic HCA-7 cells was rapidly reversible upon a switch to normoxia after 24 hours over a similar time course to that of the changes in 15-PGDH mRNA expression.
